# A Retrospective Cohort Study of Intravenous Immunoglobulin Therapy in the Acute Phase of Kawasaki Disease: The Earlier, the Better?

**DOI:** 10.1155/2021/6660407

**Published:** 2021-06-18

**Authors:** Wei Li, Xiufang He, Li Zhang, Zhouping Wang, Yanfei Wang, Huimei Lin, Jia Yuan, Xiaofei Xie, Youzhen Qin, Ping Huang

**Affiliations:** ^1^Department of Cardiology, Guangzhou Women and Children's Medical Center, Guangzhou Medical University, Guangzhou 510120, China; ^2^Department of Pediatric Cardiovascular, The First Affiliated Hospital of Sun Yat-sen University, Guangzhou 510080, China; ^3^Department of Medical Record, Guangzhou Women and Children's Medical Center, Guangzhou Medical University, Guangzhou 510120, China

## Abstract

**Background:**

Although intravenous immunoglobulin (IVIG) is expected to prevent coronary artery abnormalities of Kawasaki disease (KD) in the acute phase, the timing and effectiveness of IVIG remain to be determined. The association of timing of IVIG administration in KD patients with coronary artery abnormalities is evaluated in this cohort study.

**Methods:**

We systematically studied KD patients from two participating institutions between 2015 and 2017. To reveal the effectiveness of IVIG treatment, these patients were classified into four groups regarding the time of IVIG treatment. Primary outcome was coronary artery abnormalities by echo at diagnosis and 12 months follow-up; secondary outcomes included inflammatory markers.

**Results:**

A total of 1281 patients were included in this study. The best time of IVIG treatment cut-off values in 12 months follow-up for predicting coronary artery abnormalities was days 7.5 of illness onset. According to the best time of IVIG treatment cut-off values, all patients were classified into 4 groups. Group 1 was defined as earlier IVIG treatment administration on days ≤4 of the illness (*n* = 77). Group 2 was defined with days 5-7 (*n* = 817), group 3 with days 8-10 (*n* = 249), group 4 with days >10 (*n* = 138). A greater proportion of IVIG-resistant KD patients were group 4 than the other three groups, and there were significant differences (*p* < 0.05). The incidence of coronary artery lesions (CALs) and coronary artery aneurysms (CAAs) in group 3 and group 4 was higher than that in group 1 (*p* < 0.05) and group 2 (*p* < 0.05) during a 12-month follow-up. Additionally, the incidence of CALs in group 1 was higher than that in group 2 but without statistical significance (*p* > 0.05). The OR was significantly higher for those who started IVIG administration more than 7 days from the onset was positively associated with the occurrence of CALs (OR, 5.3; 95% CI, 2.0-13.9) and CAAs (OR, 13.5; 95% CI, 2.9-14.1) 12 months after initial onset. Multivariate regression revealed that the timing of IVIG treatment and IVIG-resistance was independent risk factors of CALs.

**Conclusions:**

IVIG treatment less than 7 days after illness onset are found to be sufficient for preventing developing coronary artery abnormalities in KD patients. Earlier IVIG treatment administration within 4 days may not increase the higher incidence of coronary artery abnormalities and IVIG resistance (Chinese Clinical Trial Registry:ChiCTR1800015800).

## 1. Introduction

It has been more than 50 years since Tomisaku Kawasaki reported the first case of mucocutaneous lymph node syndrome in Japanese children [1, 2]. Kawasaki disease (KD) occurs mainly in children, which surpasses acute rheumatic fever as a leading cause of acquired heart disease [3]. Nevertheless, KD has become a leading cause of cardiac morbidity in the world.

Intravenous immunoglobulin (IVIG) therapy can remarkably reduce the high chance of causing coronary artery abnormalities by 20-25% to less than 5% [4], which represents as great progress in KD. The definitive trial by Newburger et al. [5] showed that a single 2 g/kg dosage of IVIG is more effective at reducing the risks of developing aneurysms than the same total dose administered once per day for 4 days in 1991. The treatment of KD with IVIG proves to be one of the most cost-effective therapeutic interventions in pediatrics nowadays. Previous studies showed that high-dose IVIG is an important treatment within 10 days of illness onset and has been demonstrated effective in dramatically reducing the risk of cardiac complications [5–7]. However, debates about the risk for IVIG unresponsiveness and coronary artery lesions (CALs) in association with the earlier timing of IVIG administration of disease onset still remain [8]. On the other hand, these big cohort studies of the effects of IVIG on the cardiac complications of KD were still rare. So the purpose of this study was to investigate the timing of IVIG administration associated with the development of coronary artery abnormalities 12 months after the onset.

In this study, we systematically investigated KD patients from two institutions. By classifying them into different groups regarding the IVIG treatment time, we examined this hypothesis of IVIG therapy in the acute phase of KD: the earlier, the better.

## 2. Methods

### 2.1. Enrollment of Patients

After the present study was reviewed and approved by the Scientific Research Ethics Committee at Guangzhou Women and Children's Medical Center and the First Affiliated Hospital of Sun Yat-sen University (NO.2018032608565814), we conducted a retrospective case-control study admitted to 2 hospitals and diagnosed with KD. And we also obtained written informed consent from the parents or guardians of all the participating children.

The medical records of KD who were admitted to the two hospitals were reviewed to determine who met the criteria for inclusion in the study between January 1, 2015, and December 31, 2017. The inclusion criteria for study subjects were as follows: (1) patients were ≤18 years old and only complete and incomplete KD cases in accordance with the existing guidelines were reviewed; (2) patients of initial onset of the disease; (3) patients received IVIG treatment (2 g/kg per day) during the acute phase of illness and maintained follow-up for at least 12 months; (4) patients had serious infection, allergy, and collagen disease. Their demographic, clinical, and laboratory data echocardiography results and outcomes were extracted from the medical records. Patients were diagnosed with KD by two experienced pediatricians using the recommended universal KD criteria proposed by the American Heart Association [3]. Classic KD was diagnosed in any children with a fever that lasts longer than five days and manifested four of the following five criteria: (i) diffuse mucosal inflammation with strawberry tongue and fissured lips, (ii) bilateral nonpurulent conjunctivitis, (iii) changes of the peripheral extremities, (iv) polymorphous exanthema, and (v) unilateral cervical lymphadenopathy. Patients were categorized as having complete and incomplete KD. Incomplete KD was defined as in a child with prolonged unexplained fever, fewer than 4 of the principal clinical findings, and compatible laboratory or echocardiographic findings [9]. Patients with KD develop a recrudescent or persistent fever at least 36 hours after the end of their IVIG infusion and are termed as IVIG-resistant KD. The treatment protocol [10] included high doses of IVIG (2 g/kg) as a single infusion over 8 to 12 h along with aspirin (30–50 mg/kg per day) during the acute phase followed by 3–5 mg/kg per day for 6–8 weeks or until resolution of coronary artery abnormalities. Combined antiplatelet and anticoagulation therapy were recommended for patients with giant aneurysms.

Using the electronic medical records databases, we collected the following variables in our study: age, gender, the day of illness onset, the day of initial IVIG treatment, laboratory data, and echocardiography results. Laboratory data including white blood cell (WBC) count, neutrophils, and C-reactive protein (CRP) before and after IVIG infusion were collected for analysis. Peripheral blood was tested before IVIG treatment and after completing the initial IVIG treatment (within 3 days). We use echocardiography in the evaluation and follow-up of coronary artery abnormalities in patients. Echocardiography was performed at enrollment and 1 month, 3 months, 6 months, and 12 months after the initial onset. Echocardiography was performed and supervised by an experienced pediatric cardiographer and with appropriate transducers. Echocardiographic evaluation of coronary artery in KD patients included imaging on the left main coronary artery, anterior descending, left circumflex, right coronary artery, and posterior descending coronary arteries. We used the definition of CALs:(1)a *z*-score ≥2 , (2) internal diameter of a segment 1.5 times that of an adjacent segment (saccular or fusiform aneurysm), and (3) irregular surface and/or perivascular brightness of the coronary wall. Coronary artery aneurysms (CAAs) were classified as Z scores in this study [4, 11]: small aneurysm: ≥2.5 to <5; medium aneurysm: ≥5 to <10, and absolute dimension <8 mm; giant aneurysm: ≥10, or absolute dimension ≥8 mm.

### 2.2. Statistical Analysis

The primary outcome variables for this study were the presence or absence of coronary artery abnormalities in the acute phase and 12 months follow-up, the duration of fever, the day of IVIG administered in the acute phase of KD, and the number and proportion of IVIG resistance. In this descriptive study, means/medians and proportions were used to describe the characteristics of the study population. The laboratory parameters before and after the initial IVIG were tested using one-way ANOVA. The proportions of Coronary artery abnormalities between the groups were compared by the *χ*^2^ test with the Yates correction or Fisher's exact test. The tests used for statistical analysis were two tailed. Data are presented as box plots in 12 months follow-up of coronary artery abnormalities. The analysis of the time of IVIG treatment for the diagnosis of coronary artery abnormalities was assessed by the area under the curve (AUC), sensitivity, and specificity at the optimal cut point according to the Youden index. Multivariate regression analysis after adjusting for gender, age, duration of fever, timing of initial IVIG treatment, IVIG resistance, and laboratory parameters were used to calculate the odds ratio with a 95% confidence interval for CALs and CAAs. All tests were two sided, and *p* < 0.05 was considered significant. All statistical tests were performed with SPSS 21.0 for Windows (SPSS, Chicago, IL, USA).

## 3. Results

### 3.1. Demographic Data and Characteristics of Patient Population

Of 1328 patients diagnosed with acute KD in the study time frame at 2 hospitals, 1281 patients met the inclusion criteria were included in the study. Among the included patients, all patients received IVIG treatment (IVIG 2 g/kg for per day) once the KD diagnosis was made. The patients included in our analysis are described in Table 1. In this cohort, 816 patients (56.9%) were male, the median age was 12 months (interquartile range, 11-36 months), and 141 (11%) of 1281 patients had IVIG resistant KD and received IVIG retreatment or additional anti-inflammatory treatments for KD.

**Figure 1:**
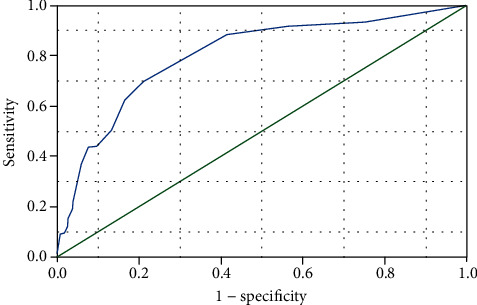
The time of IVIG treatment and the patients with coronary artery abnormalities due to Kawasaki disease in 12 months follow-up. Shown are receiver-operating-characteristic (ROC) curves for days of illness with IVIG treatment and coronary artery abnormalities in 12 months follow-up with KD cohorts. The best days of IVIG treatment cut-off values for predicting coronary artery abnormalities were 7.5 days. The area under the curve (AUC) was 0.80 (95% CI, 0.76 to 0.84).

Figure 1 shows the days of illness with IVIG treatment versus a diagnosis of coronary artery abnormalities in 12 months follow-up yielded an AUC of 0.80 (95% CI, 0.76 to 0.84, *p* < 0.001). The best time of IVIG treatment cut-off values in 12 months follow-up for predicting coronary artery abnormalities was days 7.5 of illness. This cut-off value during 12 months follow-up predicted coronary artery abnormalities with sensitivity, specificity, positive predictive value, negative predictive value, and diagnostic accuracy of 88%, 59%, 79%, 71%, and 80%, respectively. According to the best time of IVIG treatment cut-off values, all patients were classified into 4 groups in this study. Group 1 was defined as earlier IVIG treatment administration on days ≤4 of the illness (*n* = 77,  6.0%). Group 2 was defined with days 5-7 (*n* = 817, 63.8%). Group 3 with days 8-10 (*n* = 249, 19.4%). Group 4 with days >10 (*n* = 138, 10.8%).

As shown in Table 2, there were no significant differences in age ≤1 year and gender among the 4 groups. Observed higher risk of IVIG resistance was positively associated with later IVIG treatment >10 days of illness. There were a greater proportion of IVIG-resistant KD patients in group 4 than the other three groups and they were significant differences (*p* < 0.05).

Laboratory baseline data analysis shows markers of inflammation, such as WBC count, % neutrophils, and CRP in Table 3. CRP and % neutrophils were higher in group 1 and group 2 than the other two groups, and there were significant differences among the four groups before or after IVIG treatment (*p* < 0.05), suggesting inflammatory reaction is severe in KD of group 1 and group 2. On the other hand, no difference in the WBC count was observed in the four groups.

Table 4 shows the actual numbers and the proportion of coronary artery abnormalities of patients and the OR and 95% confidence intervals for each variable in relation to the development of CALs and CAAs, calculated by logistic analysis. The follow-up data indicated that 97% (1242/1281) of CALs returned to normal in terms of diameter as assessed by echocardiography in the 12 months after the illness. All of these patients had no death in the acute phase and follow-up. Furthermore, the incidence of CALs and CAAs in group 1, group 2, group 3, and group 4 at the acute phase was 14.1, 19.5, 35.3, and 58.7%; 2.6, 3.5, 8.4, and 30.4%, respectively. During 12 months follow-up, a significantly increased incidence of CALs and CAAs was observed in group 3 and group 4 compared with group 1 and group 2. The higher incidence of CALs and CAAs in group 3 and group 4 than the other two groups and significantly different in statistical analysis during 12 months follow-up (*p* < 0.05). The incidence of CALs in group 1 was higher than that in group 2 but without statistical significance (*p* > 0.05). Multivariate logistic regression analysis showed that receipt of IVIG >days 7 of illness at one month was positively associated with the occurrence of CALs (OR, 2.1; 95% CI, 1.4-3.0) and CAAs (OR, 3.9; 95% CI, 1.9-8.2). There was a significant difference in the higher proportion of CALs for those who started IVIG administration more than 7 days from the onset was positively associated with the occurrence of CALs (OR = 5.3; 95%CI = 2.0 − 13.9, *p* = 0.001) and CAAs (OR = 13.5; 95%CI = 2.9 − 14.1, *p* < 0.001) 12 months after initial onset. Multivariate regression also revealed that the timing of IVIG treatment and IVIG resistance were independent risk factors of CALs. These results also showed a significant positive correlation between the risk of coronary artery abnormalities and the time of IVIG treatment >days 7. The number and proportions of GCAAs among the four groups were low and not compared in this study.

## 4. Discussion

KD has become a leading cause of cardiac morbidity in children in many countries nowadays and the incidence of KD in children has increased over time, especially in Asia [12, 13]. KD is an acute, self-limited vasculitis of small and medium-sized arteries that leads to CALs in approximately 25% of untreated patients [14, 15]. This breakthrough was largely based on the published report by Furusho et al. [16] about the unrelated discovery of intravenous immunoglobulin as the treatment for idiopathic thrombocytopenic purpura. Based on their initial finding, IVIG appeared to decrease the incidence of coronary artery aneurysm in patients with KD. Furusho et al. [5] published a controlled study and found that a single infusion of 2,000 mg/kg per day was more effective than a regimen of 400 mg/kg per day given for 5 days. Afterwards, a high dose of IVIG has become the cornerstone of the management of acute KD for decades. Timely treatment with IVIG has decreased this risk to 3% to 5% [17]. While IVIG therapy is expensive, it is relatively safe, and the benefit typically substantially outweighs the risks [18]. IVIG is a biological product of pooled donor plasma. Adverse effects vary among products. Coombs-positive hemolytic anemia and self-resolving aseptic meningitis have been reported but are rare [19, 20]. In this work, we enrolled 1281 KD patients from the two institutions to delineate the risk for the occurrence of CALs association with the timing of IVIG treatment.

Nowadays, it is known that KD is characterized by systemic inflammation in all the medium-sized arteries during the acute febrile phase, leading to associated main clinical findings [15, 21]. It is reported that necrotizing arteritis occurred within 2 weeks after fever onset [15, 22]. It is only self-limited process and progressively destroys the arterial wall into the adventitia, causing aneurysms [23]. The present study is compatible with the idea that the decreased risk of cardiac complications among patients with KD who receive earlier IVIG treatment is due primarily to higher initial disease severity. So we attempted to observe the association between earlier treatment and cardiac complications. Numerous results suggested that IVIG treatment need not be delayed in patients diagnosed with KD. As opposed to delaying treatment for patients with KD who are diagnosed earlier, the literature suggests that more aggressive treatment of patients with high initial disease severity may help protect against cardiac complications [7]. Our study observed lower risks of cardiac complications and proportions of IVIG-resistance among patients treated with IVIG as early as possible. IVIG treatment >10 days after illness onset was considered to be prone to develop IVIG resistance and high risks of cardiac complications.

As in previous reports [24, 25], we recorded a higher risk for development to CALs if IVIG was administrated after 10 days of disease onset. A significant beneficial effect of IVIG administration prior to 10 days of fever was also identified in randomized clinical trials [26]. Because IVIG is more likely to be able to interrupt vascular inflammation in the acute stage of illness. Additionally, some studies showed none of the IVIG responder children required readministration of IVIG to decrease the risk for development of CA pathology [11, 19, 20]. Children with KD are still at risk for coronary artery abnormalities even after IVIG administration. A risk of 8% has been reported for CALs irrespective of the administration of IVIG within 8 or after 10 days of fever onset [13]. Although IVIG treatment within 10 days was recognized as an evidence-based standard, the best time for IVIG treatment with respect to the fever onset is still debatable. Our study showed that 7 days of fever were identified as an appropriate cut-off point for IVIG administration. It is also showed that patients treated within day 7 of illness onset may develop CALs and CAAs significantly less frequently as compared to those who are treated on days ≥8. We found the low-risk level for occurrence or almost complete resolution of CA abnormalities in children with KD with respect to the time of IVIG administration within 7 days of fever onset. Therefore, late IVIG is less effective for preventing CALs. Patients whose symptoms do not meet the classic KD criteria are problematic, and the diagnosis is frequently delayed. However, it is difficult to determine the effectiveness of IVIG after 7 days based only on coronary outcomes.

The risk factors of IVIG resistance as well as CALs and coronary aneurysms have been extensively studied [10, 27]. About one-third of patients with KD rapidly develop symptoms and are diagnosed within day 4 of illness in Japan [28, 29]. They are recognized as high risk of IVIG resistance and coronary artery abnormalities [30]. The American Heart Association scientific statement to KD treatment recommend [4] that IVIG should be administered to children as early as the diagnosis, even presenting after the 10th day of illness if they have either persistent fever without another explanation or aneurysms and ongoing systemic inflammation. It is reported that a lower rate of IVIG treatment resistance within day 5 suggested the possibility that early-administered IVIG is more likely to subside inflammation effect [31]. Similarly, in our study, the results showed IVIG treatment within day 4 of illness was reported to be not associated with increasing IVIG resistance and risk of developing coronary artery abnormalities. Some studies speculated that early IVIG treatment might have resulted in an earlier effect in immunoglobulins levels change during the acute phase of KD [32–34]. Similarly, some research investigated the precise pharmacokinetics of IVIG as well as the influence of immunological changes during the acute phase of KD on treatment response [23, 33, 35–39]. The optimal timing of the initial IVIG in these patients has long been discussed to improve their outcomes [17]. In our study, the proportion of IVIG resistance was 13.0% in group 1. In line with other results, approximately 10% of patients are resistant to IVIG [40, 41], although our results do not provide a definitive answer to the question of when to administer the initial IVIG in early-diagnosed KD. Patients after early IVIG treatment would be preferable to closely monitored for resistance. There have been no clinical trials comparing the effectiveness of IVIG with different days of illness onset. At the end of the midterm follow-up, no fatal case occurred. Additional medical therapies including antiplatelet therapy in patients with KD are administered to reduce the prevalence of CALs, reduce systemic inflammation, and prevent coronary thrombosis [42]. If CAAs are already present, antiplatelet and anticoagulation therapy is combined to reduce the risk of adverse cardiovascular events and to prevent coronary thrombosis [43, 44].

This was a retrospective study performed by analyzing medical records and clinic visits. Enrollment of KD patients was based on standard IVIG treatment and midterm follow-up, inevitably introducing selection bias. Because of the limitations of this study, future studies are needed.

## 5. Conclusions

IVIG treatment less than 7 days of illness onset is found to be sufficient for preventing developing coronary artery abnormalities in KD patients. Earlier IVIG treatment administration within days 4 may not increase the higher incidence of coronary artery abnormalities and IVIG resistance.

## Figures and Tables

**Table 1:** Baseline characteristics of 1281 Kawasaki disease patients.

Variables	No. (%)
No. of patients	1281
Age median (IQR), months	12 (11-36)
Age ≤1 y, no. (%)	717 (56.0)
Gender (male : female)	1.75 : 1 (816 : 465)
Days of first hospital visit (d)	6 (5-7)
Illness days of initial IVIG (d)	6 (5-8)
Total hospital stay (d)	3 (2-4)
IVIG-resistant KD, no. (%)	141 (11.0)

Expressed as the median (interquartile range: IQR), or *n* %. IVIG: intravenous immunoglobulin.

**Table 2:** The characteristics in 1281 patients in 4 groups with Kawasaki disease.

Patient characteristic	Group 1 (*n* = 77)	Group 2 (*n* = 817)	Group 3 (*n* = 249)	Group 4 (*n* = 138)	*p* value
Age (months)	12 (7-24)	12 (12-36)	12 (11.5-36)	12 (8-24)	0.009
Means ± SD	17.8 ± 15.7	23.2 ± 19.7	26.4 ± 23.4	22.3 ± 22.0	
Age ≤1 yr.	50 (64.9)	450 (55.1)	133 (53.4)	84 (60.9)	0.189
Gender (male : female)	1.4 : 1	1.7 : 1	1.8 : 1	2.2 : 1	0.464
Days of first hospital visit (d)	4 (3-4)	5 (5-6)	8 (7-9)	13 (11-15)	—
Illness days of initial IVIG (d)	4 (4-4)	6 (5-7)	9 (8-9)	13.5 (12-16)	—
Total hospital stay (d)	3 (2-5)	2 (2-3)	2 (2-4)	4 (2-7)	<0.001
IVIG-resistant KD cases	10 (13.0)	79 (9.7)	27 (10.8)	25 (18.1)	0.03

Data are presented as median (interquartile range) or *n* (%). IVIG: intravenous immunoglobulin. KD: Kawasaki disease.

**Table 3:** The laboratory parameters in 1281 patients in 4 groups with Kawasaki disease.

	Laboratory parameter	Group 1 (*n* = 77)	Group 2 (*n* = 817)	Group 3 (*n* = 249)	Group 4 (*n* = 138)	*p* value
Baseline data	WBC count (10^3^/mm^3^)	15.5 ± 6.1	15.9 ± 5.5	16.2 ± 6.6	15.9 ± 7.3	0.764
	Neutrophils (%)	62.0 ± 14.7	61.7 ± 14.6	60.6 ± 13.9	55.2 ± 14.8	<0.001
	CRP (mg/L)	82.7 ± 55.2	89.4 ± 60.7	77.9 ± 57.5	67.1 ± 56.3	<0.001
Data after treatment	WBC count (10^3^/mm^3^)	11.0 ± 4.4	9.97 ± 4.24	9.7 ± 4.6	9.6 ± 4.2	0.095
	Neutrophils (%)	37.5 ± 15.3	38.5 ± 15.3	41.1 ± 16.0	36.8 ± 16.6	0.039
	CRP (mg/L)	43.1 ± 47.1	51.9 ± 52.6	36.6 ± 40.3	29.5 ± 40.1	<0.001

Expressed as means ± SD. CRP: C-reactive protein; WBC: white blood cell.

**Table 4:** Coronary artery abnormalities for patients with Kawasaki disease in 4 groups during 12 months follow-up.

	Cardiac complications		Acute phase	1 month	3 months	6 months	12 months
Group 1 (*n* = 77)	CALs		11 (14.3)	9 (11.7)	6 (7.8)	2 (2.6)	1 (1.3)
	CAAs		2 (2.6)	2 (2.6)	1 (1.3)	1 (1.3)	—
	GCAAs		1 (1.3)	1 (1.3)	1 (1.3)	—	—
Group 2 (*n* = 817)	CALs		159 (19.5)	90 (11.0)	31 (3.8)	10 (1.2)	7 (0.9)
	CAAs		29 (3.5)	14 (1.7)	6 (0.7)	5 (0.6)	2 (0.2)
	GCAAs		3 (0.4)	2 (0.2)	2 (0.2)	1 (0.1)	—
Group 3 (*n* = 249)	CALs		88 (35.3)	51 (20.5)	23 (9.2)	15 (6.0)	11 (4.4)
	CAAs		22 (8.8)	16 (6.4)	13 (5.2)	10 (4.0)	8 (3.2)
	GCAAs		2 (0.8)	2 (0.8)	3 (1.2)	3 (1.2)	3 (1.2)
Group 4 (*n* = 138)	CALs		81 (58.7)	56 (40.6)	38 (27.5)	29 (21.0)	20 (14.5)
	CAAs		42 (30.4)	30 (21.7)	21 (15.2)	20 (14.5)	13 (9.4)
	GCAAs		6 (4.3)	8 (5.8)	8 (5.8)	5 (3.6)	5 (3.6)
*p* value	CALs	Group 1 vs. 2	0.269	0.857	0.087	0.277	0.515
		Group 2 vs. 3	<0.001	<0.001	0.001	<0.001	0.001
			OR 2.3 95% CI 1.7-3.1	OR 2.1 95% CI 1.4-3.0	OR 2.6 95% CI 1.5-4.5	OR 5.2 95% CI 2.3-11.7	OR 5.3 95% CI 2.0-13.9
	CAAs	Group 1 vs. 2	0.491	0.407	0.469	0.418	—
		Group 2 vs. 3	0.001	<0.001	<0.001	<0.001	<0.001
			OR 2.7 95% CI 1.5-4.7	OR 3.9 95% CI 1.9-8.2	OR 7.4 95% CI 2.8-19.8	OR 6.8 95% CI 2.3-20.1	OR 13.5 95% CI 2.9-14.1

Expressed as *n* (%). CALs: coronary artery lesions; CAAs: coronary artery aneurysms; GCAAs: giant coronary artery aneurysms. OR: odds ratio.

## Data Availability

All data relevant to the study are included in the article.
